# Association of Adverse Childhood Experiences With Frailty Index Level and Trajectory in China

**DOI:** 10.1001/jamanetworkopen.2022.25315

**Published:** 2022-08-01

**Authors:** Qing Wang

**Affiliations:** 1 Department of Biostatistics, School of Public Health, Cheeloo College of Medicine, Shandong University, Jinan, Shandong, China; 2 National Institute of Health Data Science of China, Shandong University, Jinan, Shandong, China

## Abstract

This cross-sectional study explores the association of adverse childhood experiences and frailty index among community-living adults aged at least 45 years in China.

## Introduction

Adverse childhood experiences (ACEs) are recognized as an important public health issue.^1^ The most widely used ACE scale, the Kaiser Permanente ACE Study, includes 10 items across domains of child abuse, neglect, and household dysfunctions. It is generated based on a sample of primarily White and educated individuals.^2^ Recently, a set of expanded ACEs (eg, socioeconomic [SE] hardships, negative peer relationships, and community-level ACEs) has been measured to understand their health implications.^1^ However, the results differ by sample population. For example, a prospective cohort study in the UK showed no difference in the association between ACEs and health outcomes across SE groups, whereas in China, childhood SE status was significantly associated with later health.^2,3^ Thus, in terms of health, what constitutes ACEs remains unknown in a specific context, which was thought to be shaped by social contexts.^4^ Because China is a unique case owing to Confucian culture and massive social shifts, this study attempts to explore health-based ACEs based on the association between 18 ACEs and frailty index (FI) in China.^5^

## Methods

This population-based cross-sectional study used the China Health and Retirement Longitudinal Study (CHARLS) data, providing a wide range of information from SE status to health conditions of community-living adults aged at least 45 years.^6^ After excluding missing values, those participated in the CHARLS surveys 2011, 2013, 2015, and 2018, and life history survey in 2014 were included. This study followed the Strengthening the Reporting of Observational Studies in Epidemiology (STROBE) reporting guideline and is not subject to ethical approval according to the London School of Economics and Political Science research ethics policy.

Individual ACEs, covering intrafamilial aggression, family dynamic, SE deprivation, and neighborhood quality, were obtained from a life history survey. Based on 41 measurements collected from CHARLS 2011, 2013, 2015, and 2018, FI was calculated and categorized as robust, prefrail, and frail, with its trajectories classified as stable at robust and prefrail, and rapidly rising to frail.^4^

Weighted generalized ordered logistic and logistic models were applied adjusting for sociodemographic factors and health behaviors in adulthood. Two-tailed *P* < .05 indicated statistical significance, with OR and 95% CIs reported. Data analysis was performed from December 1 to 30, 2021. Stata version 15 (StataCorp LLC), was used. See eMethods in the Supplement for details.

## Results

Among the 11 568 respondents (mean [SD] age, 57.95 [9.16] years; 6230 female respondents [54%]), 1869 (16%) reported frail status, with 2342 (20%) having rapidly rising to frail trajectory (Table 1). An increase in the number of ACEs was associated with a 20% (odds ratio [OR], 1.20; 95% CI, 1.16-1.23) and 19% (OR, 1.19; 95% CI, 1.16-1.23) increase in the likelihood of being in frail status, and rapidly rising trajectory. Loss or threat of loss within the family (eg, household mental or serious illness, serious childhood illness or injury), socioeconomic deprivation (excluding child hunger), low-quality neighbors, and peer bullying victimization were related to FI, whereas the prevalence of exposure to family dynamics (1154 of the 11 568 respondents) was less than 10%, and not significantly associated with FI. After adjustments of co-occurring ACEs, a limited association of intrafamilial aggression was found, which may be due to the prevailing views of stricter parenting practices in China (Table 2).

**Table 1.** Participant Descriptive Statistics Before and After Imputation and Weighting

Characteristic	Before imputation and weighting, No. (%)	After imputation and weighting, %
	Total sample (n = 11568)	
FI categories		
Robust (frailty index ≤0.10)	1725 (14.91)	17.44
Prefrail (>0.10 to <0.25)	7974 (68.93)	67.60
Frail (frailty index ≥0.25)	1869 (16.16)	14.96
FI trajectory		
Stable at robust or prefrail	9206 (79.58)	80.99
Rapidly rising to frail	2362 (20.42)	19.01
FI for stable at robust or prefrail group, mean (SD)		
FI in 2011	0.13 (0.05)	0.13 (0.001)
FI in 2013	0.14 (0.05)	0.14 (0.001)
FI in 2015	0.14 (0.06)	0.14 (0.001)
FI in 2018	0.15 (0.05)	0.15 (0.001)
FI for rapidly rising to frail group, mean (SD)		
FI in 2011	0.25 (0.09)	0.25 (0.002)
FI in 2013	0.30 (0.10)	0.29 (0.002)
FI in 2015	0.33 (0.11)	0.33 (0.003)
FI in 2018	0.34 (0.12)	0.35 (0.003)
No. of ACEs, mean (SD), No.	3.26 (1.77)	3.17 (0.02)
Age in 2011, mean (SD), y	57.95 (9.16)	58.47 (0.11)
Female	6230 (53.86)	52.24
Male	5338 (46.14)	47.76
Ever being unmarried from 2011 to 2018	2634 (22.77)	23.68
Education		
Illiterate	3262 (28.20)	25.32
Elementary school	2129 (18.40)	17.02
Junior high school	2544 (21.99)	21.70
High school or above	3633 (31.41)	35.96
Ever being heavy drinker	2921 (25.25)	24.64

Abbreviations: ACE, adverse childhood experience; FI, frailty index.

**Table 2.** Association of ACE With FI Categories and Trajectories Using Imputed Data: Weighted Generalized Ordered Logistic Model and Logistic Model

Characteristic	Total sample^a^, No. (%) (N = 11 568)	Effect size for association, OR (95% CI)^b^		
		FI categories^c^		FI trajectories^d^
		Robust vs prefrail	Prefail vs frail	
Association between cumulative score of ACEs and FI				
ACEs, mean (SD), No.	3.26 (1.77)	1.19 (1.14-1.25)	1.19 (1.15-1.23)	1.20 (1.16-1.24)
Association between types of individual ACEs and FI				
Loss or threat of loss within the family				
Household mental illness	420 (3.63)	1.37 (0.84-2.02)	1.57 (1.18-2.07)	1.57 (1.20-2.06)
Severe illness in family	2449 (21.17)	1.37 (1.11-1.70)	1.52 (1.31-1.77)	1.50 (1.30-1.73)
Parental emotion issue	3949 (34.14)	1.20 (0.99-1.47)	1.05 (0.92-1.21)	1.09 (0.95-1.25)
Serious childhood illness or injury	1001 (8.65)	1.37 (1.03-1.81)	1.67 (1.34-2.08)	1.66 (1.36-2.03)
Childhood socioeconomic status				
Low parental education	6079 (52.55)	1.33 (1.14-1.55)	1.22 (1.07-1.39)	1.28 (1.13-1.45)
Parental unemployment	1154 (9.98)	1.33 (1.03-1.71)	1.14 (0.94-1.41)	1.08 (0.88-1.31)
Family financial problems	4652 (40.21)	1.23 (1.03-1.47)	1.28 (1.12-1.46)	1.36 (1.20-1.54)
Childhood hunger	3319 (28.69)	0.74 (0.63-0.86)	0.71 (0.70-0.95)	0.81 (0.70-0.94)
Childhood intrafamilial aggression				
Parental physical maltreatment	3186 (27.54)	1.12 (0.92-1.37)	1.10 (0.95-1.27)	1.11 (0.97-1.28)
Emotional neglect	3949 (34.14)	1.14 (0.98-1.33)	1.09 (0.94-1.25)	1.08 (0.95-1.23)
Sibling aggression victimization	692 (5.98)	1.24 (0.88-1.75)	1.09 (0.86-1.38)	1.09 (0.87-1.36)
Witnesses of inter-parental violence	877 (7.58)	1.19 (0.89-1.40)	1.12 (0.90-1.40)	1.15 (0.93-1.41)
Family dynamics				
Poor parent-child relationship	204 (1.76)	1.41 (0.57-3.50)	1.49 (0.94-2.36)	1.50 (0.97-2.33)
Parental separation or divorce	100 (0.86)	1.28 (0.47-3.50)	1.37 (0.77-2.45)	1.50 (0.88-2.55)
Household substance abuse	802 (6.93)	1.17 (0.86-1.60)	1.13 (0.89-1.42)	1.09 (0.87-1.36)
Household criminality	128 (1.11)	0.73 (0.26-2.02)	0.96 (0.59-1.56)	0.80 (0.49-1.30)
Neighborhood quality				
Low-quality neighbors	5470 (47.29)	1.22 (1.07-1.38)	1.22 (1.07-1.38)	1.21 (1.08-1.37)
Peer bullying victimization	1723 (14.89)	1.07 (0.86-1.37)	1.19 (1.00-1.41)	1.12 (0.98-1.29)

Abbreviations: ACE, adverse childhood experience; FI, frailty index.

^a^ Includes 11 568 observations for each effect size.

^b^ Eighteen ACEs were conceptualized as a cumulative score based on the total number of ACEs experienced and as individual ACE types. The 18 individual ACEs were controlled simultaneously when examining the associations between types of ACEs and FI. Weighted generalized ordered logistic model and weighted logistic model were applied using imputed data with demographic characteristics (sex, marital status, and age), educational attainment, and ever being a heavy drinker controlled. The weight of the longitudinal data was calculated using the sample attrition adjustment method to correct the attribution bias in the cohort.

^c^ Three categories of FI were defined: robust (frailty index ≤0.10), prefrail (frailty index >0.10 to <0.25), and frail (frailty index ≥0.25).

^d^ FI trajectories were classified into 2 groups (eg, stable at robust and prefrail, rapidly rising and frail) using group-based trajectory modeling.

## Discussion

Cumulative ACEs were associated with increased frailty events and a faster decline in FI in their middle and older age. However, the associations of different types of adversities were heterogeneous. Older Chinese people were sensitive to expanded ACEs including socioeconomic deprivation (excluding child hunger), low-quality neighbors, and peer bullying in FI.

Limitations of the study include retrospective self-evaluations, and the study did not include some common ACEs (eg, sex abuse) and identify all the health-based ACEs. Our findings should be applied to other countries with caution.

## Notes

Supplement. **eMethods.** Details on Methodology and Sample
