# Supplementary material for: Association of Adverse Childhood Experiences With Frailty Index Level and Trajectory in China
Source: JAMA Netw Open. 2022 Aug 1;5(8):e2225315. doi: 10.1001/jamanetworkopen.2022.25315 (PMC9344355; doi:10.1001/jamanetworkopen.2022.25315)
Supplement: Supplement. — eMethods. Details on Methodology and Sample [file jamanetwopen-e2225315-s001.pdf]

Supplemental Online Content

Wang Q. Association of adverse childhood experiences with frailty index level and trajectory in China. *JAMA Netw Open.* 2022;5(8):e2225315. doi:10.1001/jamanetworkopen.2022.25315

eMethods. Details on Methodology and Sample

This supplemental material has been provided by the authors to give readers additional information about their work.

© 2022 Wang Q et al. *JAMA Network Open*.

Supplemental methods. Details on Methodology and Sample

Data source

This cross-sectional study was conducted using the China Health and Retirement Longitudinal Study (CHARLS) data, which is a nationally representative sample of people 45 years and older. A more detailed description of the study design and sampling procedure can be found in the cohort profile of CHARLS.[1] This study follows the Strengthening the Reporting of Observational Studies in Epidemiology (STROBE) reporting guideline for cross-sectional studies. The first CHARLS nationwide data were collected in July 1 to September 30, 2011, December 2011 and January 2012, covering an extensive range of information, such as demographic characteristics, socioeconomic status, and health status. The CHARLS cohort was followed up every 2 years, with new sample members recruited to ensure representative. The second, third and fourth waves were conducted from July 1 to September 30 in 2013, 2015 and 2018, respectively. The panel response rates were higher than 85%. There were 13567 respondents participating in the four waves. The CHARLS life history survey, conducted from June 1 to December 31, 2014, retrospectively collected the life history information of all live respondents (including adverse childhood experiences (ACEs)) in the previous waves. We successfully conducted 1:1 matching of 12748 respondents who had completed CHARLS 2011-2018 and life history surveys with demographic information. After exclusion of 1029 participants with missing information on ACE components, and 141 participants without any data on adulthood health status, 11 568 participants with complete data

© 2022 Wang Q et al. *JAMA Network Open*

were included. Data analysis was performed from December 1 to 30, 2021. As robust check, incomplete data were imputed with the multiple imputation method by chained equations.

Adverse Childhood Experiences

We conceptualized 18 ACEs as a cumulative score based on the total number of ACEs experienced and as individual ACE types.[2] Self-reported ACEs, including intrafamilial aggression and neglect, family dynamics, loss or threat of loss within the family, socioeconomic deprivation, and neighborhood quality, were collected through a bunch of questionnaires in CHARLS life history survey of 2014. Responses to each item were dichotomized as yes (1) or no (0), and then summed to generate a cumulative ACE score for each participant, ranging from 0 to 18.

© 2022 Wang Q et al. *JAMA Network Open.*

Appendix Table 1. Questionnaire Items of Each ACE Indicator

Types of ACE	Domain	Questionnaire Items	Standard
**Childhood intrafamilial aggression**	**Parental physical maltreatment**	When you were growing up, did your women/men guardian ever hit you? 1. often, 2. sometimes, 3. rarely; 4. never	1,2
	**Sibling aggression victimization**	When you were growing up, did your siblings ever hit you? 1. often, 2. sometimes, 3. rarely; 4. never	1,2
	**Emotional neglect**	"How would you rate your relationship with your women/ men guardian when you were growing up? 1. very good, 2. good, 3. fair, 4. poor"; "How much love and affection did your women guardian give you? 1. often, 2. sometimes, 3. rarely; 4. never"; "How much effort did your women guardian put into watching over you? 1. a lot, 2. some, 3. a little, 4. none"; "How strict was your women/men guardian with the rules for you? 1. not at all strict; 2. a little strict; 3. somewhat strict; 4. very strict"; "Did your women/men guardian treat your siblings better than you? 1. not at all; 2. a little; 3. somewhat; 4. very	Respondents responded 3 or 4 to either of the following questions
	**Witness of inter-parental violence**	When you were growing up, did your parents often beat up each other? 1. often, 2. sometimes, 3. rarely; 4. never	1,2
**Childhood socioeconomic status**	**Low parental education**	The father and mother's educational attainments were both categorized as illiterate	Illiterate
	**Parental unemployment**	When you were growing up, men or women guardians did not work for pay or work in a family business	
	**Family financial problems**	Respondents were asked to classify household financial status before his or her 17 years old	Worse than others
	**Childhood hunger**	When you were a child before age 17 was there ever a time when your family did not have enough food to eat?	Yes
**Family dynamics**	**Household substance abuse**	During the years you were growing up, did your women/men guardian ever have alcoholism or drug?	Yes

© 2022 Wang Q et al. *JAMA Network Open.*

	**Parental separation or divorce**	Were your biological parents divorced (including long separation due to emotional problems) before you were 17 years?	Yes
	**Poor parent-child relationship**	How would you rate your relationship with your women/men guardian when you were growing up? 1. Excellent, 2. Very good,3. Good,4. Fair,5. Poor	Poor to women or men guardian
	**Household criminality**	During the years you were growing up, have your women/men guardian ever been arrested or sent to prison?	Yes
**Loss or threat of loss within the family**	**Household mental illness**	Did your women/men guardian have abnormality of mind when you were young?	Yes
	**Parental emotional issue**	During your childhood did your women/men guardian often feel nervous and anxious?<br>During your childhood did your women/men guardian get upset easily or feel panicky?<br>During the years you were growing up, had your men guardian showed continued signs of sadness or depression that lasted 2 weeks or more? 1. A little of the time, 2. Some of the time, 3. Good part of the time, 4. Most of the time	3, or 4 to either of the two questions
	**Severe illness in family**	Did your women/men guardian have a long time being sick on bed when you were young?<br>Did your women/men guardian have a serious deformity when you were young?	Yes to either of the two questions
	**Serious childhood illness or injury**	Before you were 15 years old (including 15 years old), would you say that compared<br>to other children of the same age,1. Much healthier,2. Somewhat healthier,<br>3. About average, 4. Somewhat less healthy, 5. Much less healthy	4,5
**Neighborhood quality**	**Low-quality neighbors**	Neighborhood quality was assessed through 4 items: safe, willing to help each other, clean, and close-knit. Responses to the questions were encoded from 1 to 4 1: yes; 2: somewhat; 3: seldom; 4: not at all. e, with summed scores ranging from 4-16.	Neighbors were divided into well or poor based on the mean score.
	**Peer bullying victimization**	When you were a child, how often were you picked on or bullied by kids in your	1, or 2 to either of the

© 2022 Wang Q et al. *JAMA Network Open.*

		neighborhood?1. often, 2. sometimes, 3. rarely; 4. never When you were a child, how often were you picked on or bullied by kids in your school? 1. often, 2. sometimes, 3. rarely; 4. never	two questions

ACEs: Adverse childhood experiences

© 2022 Wang Q et al. *JAMA Network Open.*

Frailty Index

Forty-one indicators were collected from CHARLS 2011, 2013, 2015 and 2018 to calculate the FI. Each deficit was categorized or mapped into the 0·00–1·00 interval, with 0·00 indicating the absence of a deficit and 1·00 indicating the maximal expression of the deficit. The FI was calculated for each respondent as the number of deficits present in a person divided by the total number of deficits.[3] Following previous literature, we did not assign weights to individual indicators that were interlinked with each other.[3] The FI is a continuous variable that ranges from 0·00 to 1·00, with a higher value indicating a worse, frailer status. Based on FI in 2011, 2013, 2015 and 2018, the average score of FI for each respondent was calculated in the study period, and was categorized as robust (≤0.10), prefrail (>0·10 to <0.25), and frail(≥0.25).

© 2022 Wang Q et al. *JAMA Network Open.*

Appendix Table 2. Questionnaire Items of Frailty Index

Definition	Coding of variables
Self-reported diagnosis of hypertension by a doctor, self-reported use of antihypertension drugs, systolic blood pressure measured to be ≥140 mm Hg, or diastolic blood pressure measured to be ≥90 mm Hg	Yes=1·00; no=0·00
Self-reported diagnosis of dyslipidemia by a doctor	Yes=1·00; no=0·00
Self-reported diagnosis of chronic lung diseases by a doctor	Yes=1·00; no=0·00
Self-reported diagnosis of liver diseases by a doctor	Yes=1·00; no=0·00
Self-reported diagnosis of heart attack, coronary heart disease, angina, congestive heart failure, or other heart problems by a doctor	Yes=1·00; no=0·00
Self-reported diagnosis of stroke by a doctor	Yes=1·00; no=0·00
Self-reported diagnosis of stomach or other digestive diseases by a doctor	Yes=1·00; no=0·00
Self-reported diagnosis of emotional, nervous, or psychiatric problems by a doctor	Yes=1·00; no=0·00
Self-reported diagnosis of asthma by a doctor	Yes=1·00; no=0·00
Self-reported diagnosis of memory-related disease by a doctor	Yes=1·00; no=0·00
Self-reported diagnosis of diabetes	Yes=1·00; no=0·00
Self-reported diagnosis of cancer by a doctor	Yes=1·00; no=0·00
Self-reported diagnosis of chronic kidney disease by a doctor	Yes=1·00; no=0·00
Cognitive impairments (MMSE)	<10, 1.00;11-17, 0.75;18-20,0.50; 21-24,0.25, >=25,0.00
Depressive symptoms (the CES-D scale)	>=12,1.00; otherwise, 0.00
Do you have any difficulty with running or jogging about 1 Km?	Yes, I have difficulty and need help, or I can not do it,1·00; otherwise, 0·00
Do you have difficulty with walking 1 km?	Yes, I have difficulty and need help, or I can not do it,1·00; otherwise, 0·00
Do you have difficulty with walking 100 metres?	Yes, I have difficulty and need help, or I can not do it,1·00; otherwise, 0·00
Do you have difficulty with getting up from a chair after sitting for a long period?	Yes, I have difficulty and need help, or I can not do it,1·00; otherwise, 0·00
Do you have difficulty with climbing several flights of stairs without resting?	Yes, I have difficulty and need help, or I can not do it,1·00; otherwise, 0·00
Do you have difficulty with stooping, kneeling, or crouching?	Yes, I have difficulty and need help, or I can not do it,1·00; otherwise, 0·00
Do you have difficulty with reaching or extending your arms above shoulder level?	Yes, I have difficulty and need help, or I can not do it,1·00; otherwise, 0·00
Do you have difficulty with lifting or carrying weights	Yes, I have difficulty and need help, or I can

© 2022 Wang Q et al. *JAMA Network Open.*

over 10 jin?	not do it,1·00; otherwise, 0·00
Do you have difficulty with picking up a small coin from a table?	Yes, I have difficulty and need help, or I can not do it,1·00; otherwise, 0·00
Because of health and memory problems, do you have any difficulty with dressing?	Yes, I have difficulty and need help, or I can not do it,1·00; otherwise, 0·00
Do you have any difficulty with bathing or showering?	Yes, I have difficulty and need help, or I can not do it,1·00; otherwise, 0·00
Do you have any difficulty with eating, such as cutting up your food?	Yes, I have difficulty and need help, or I can not do it,1·00; otherwise, 0·00
Do you have any difficulty with getting into or out of bed?	Yes, I have difficulty and need help, or I can not do it,1·00; otherwise, 0·00
Do you have any difficulties with using the toilet, including getting up and down?	Yes, I have difficulty and need help, or I can not do it,1·00; otherwise, 0·00
Do you have any difficulties with controlling urination and defecation?	Yes, I have difficulty and need help, or I can not do it,1·00; otherwise, 0·00
Do you have any difficulties with doing household chores?	Yes, I have difficulty and need help, or I can not do it,1·00; otherwise, 0·00
Do you have any difficulties with preparing hot meals?	Yes, I have difficulty and need help, or I can not do it,1·00; otherwise, 0·00
Do you have any difficulties with shopping for groceries?	Yes, I have difficulty and need help, or I can not do it,1·00; otherwise, 0·00
Do you have any difficulties with managing your money	Yes, I have difficulty and need help, or I can not do it,1·00; otherwise, 0·00
Do you have any difficulties with taking medications?	Yes, I have difficulty and need help, or I can not do it,1·00; otherwise, 0·00
Do you have one of physical or brain damage/intellectual disabilities?	Yes=1·00; no=0·00
Is your hearing very good, good, fair, poor, or very poor?	Very poor, 1.00; poor, 0.75; fair,0.5; good,0.25; very good,0
How good is your eyesight for seeing things at a distance?	Very poor, 1.00; poor, 0.75; fair,0.5; good,0.25; very good,0
How good is your eyesight for seeing things up close?	Very poor, 1.00; poor, 0.75; fair,0.5; good,0.25; very good,0
Would you say your health is very good, good, fair, poor or very poor?	Very poor, 1.00; poor, 0.75; fair,0.5; good,0.25; very good,0
Think about your life-as-a-whole. How satisfied are you with it?	Very poor, 1.00; poor, 0.75; fair,0.5; good,0.25; very good,0

Then, using group-based trajectory modelling, the FI trajectories for all respondents was classified into two groups, namely, stable at robust or prefrail, and highly

© 2022 Wang Q et al. *JAMA Network Open.*

increasing to frail status, reflecting the temporal variation in FI from 2011 to 2018. Maximum likelihood estimation was used to estimate the parameters of the model and identify clusters of individuals with similar trajectories while assuming the concentration data distributed as the censored normal distribution. Analyses were conducted using Traj macro in STATA. The number of trajectory groups that best fit the data was determined using the lower BIC values, the accuracy of the classification (the average posterior probability (AvePP ≥0.80), and the overall adequacy and interpretability of the model (≥5% of participants were assigned to each trajectory grouping) of our participants were also considered.[4] Each participant was assigned to the group for which their average posterior probability was greatest. Based on BIC, and AvePP, the two groups for trajectory of FI were selected. The two groups were classified as stable at robust or prefrail, and highly increasing to frail status.

Appendix table 3. Fit Information for the Group-based Trajectory Model

Groups	AIC	BIC	SSA-BIC	Entropy	Percent(%)
2	55894.74	55868.26	55872.42	0.905	79.93%/20.07%
3	59470.6	59430.88	59437.12	0.885	67.72%/27.18%/5.10%
4	60567.01	60514.05	60522.36	0.847	28.15%/9.83%/59.478%/2.55%
5	61079.03	61012.83	61023.22	0.744	27.86%/3.87%/54.34%/12.97%/0.95%

Statistical analysis

Participant descriptive statistics before and after imputation and weighting were calculated. Weighted generalized ordered logistic model and weighted logistic model were constructed to examine ACEs association with FI and its trajectory at a later age. ACEs cumulative score and types, were regressed respectively. The 18 individual ACEs were controlled simultaneously when examining the associations between types

© 2022 Wang Q et al. *JAMA Network Open*.

of ACEs and frailty, so we could understand whether effect sizes reflect an effect of certain ACEs, or effects of other co-occurring ACEs. Demographic characteristics, socioeconomic status, and health behaviors in adulthood were selected and controlled for Stepwise Regression procedure. Adulthood socioeconomic status was measured by educational attainment (a category variable: illiterate, elementary school, junior high school, and high school or above). Demographic variables included sex, marital status (unmarried included ever being single, divorced, or separated in the studied period), and age. Ever being a heavy drinker was adjusted for health behaviors. Judging from Brant Test, the parallel-lines assumption is violated, with *chi(2)* 27.10(*P* value:0.001] and 66.69(*P* value: 0.000) for association of number and types of ACEs with FI level. Generalized ordered logistic regression, which relaxes the proportionality assumptions, allows both the intercepts and the slope to vary across the categories of the outcome variable.[5] Generalized ordered logistic model was used to estimate the association of number and types of ACEs with FI level.[6] Regarding the association between ACEs and FI trajectory, logistic models were applied.

Since individuals with continued participation over years might be different from those who leave the cohort, attrition could lead to potential bias. The sample attrition adjustment method was applied to obtain the weight of our longitudinal data, and weighted regression models with robust variance estimates were derived from generalized estimating equations. Because 719 participants did not participating in the life history survey, the final weights further accounted for the response probability using the inverse predicted probability. Two-tailed $P < .05$ indicated statistical

© 2022 Wang Q et al. *JAMA Network Open.*

significance. Coefficients or Odds ratios (ORs) and 95% CIs were reported. STATA, version 15 (StataCorp LLC), was used for all calculations.

References

1. Zhao Y, Hu Y, Smith JP, Strauss J, Yang G. Cohort profile: the China Health and Retirement Longitudinal Study (CHARLS). *Int J Epidemiol*. 2014;43(1):61-68.

2. Ports KA, Ford DC, Merrick MT, Guinn AS. *ACEs: Definitions, measurement, and prevalence,* IN Asmundson GJ, Afifi TG. Adverse Childhood Experiences. Academic Press, 2020; 17-34.

3. Fan J, Yu C, Guo Y, et al. Frailty index and all-cause and cause-specific mortality in Chinese adults: a prospective cohort study. *Lancet Public Health.* 2020; 5: e650–60.

4. Sakuma A, Ueda I, Shoji W, Tomita H, Matsuoka H, Matsumoto K.Trajectories for Post-traumatic Stress Disorder Symptoms Among Local Disaster Recovery Workers Following the Great East Japan Earthquake: Group-based Trajectory Modeling. *J Affecti Disorders*. 2020; 274; 742-751.

5. Long JS, Freese J. *Regression Models for Categorical Dependent Variables Using Stata*. Stata Press College Station, TX.2006.

6. Williams R. Generalized ordered logit/partial proportional odds models for ordinal dependent variables. *Stata J.* 2006; 6: 58–82.

© 2022 Wang Q et al. *JAMA Network Open.*
